# Novel Medical Therapy of Cesarean Scar Pregnancy With a Viable Embryo Combining Multidose Methotrexate and Mifepristone: A Case Report

**DOI:** 10.1097/MD.0000000000001697

**Published:** 2015-10-16

**Authors:** Emmanouil Kalampokas, Ioannis Boutas, Konstantinos Panoulis, Theodoros Kalampokas

**Affiliations:** From the Second Department of Obstetrics and Gynecology, Aretaieion Hospital, University of Athens Medical School, Athens, Greece.

## Abstract

An ectopic pregnancy is defined as cesarean scar pregnancy (CSP) when the products of conception are implanted within the myometrium in the area of a cesarean section scar. CSP can be a life-threatening condition and to date there is no clear consensus for CSP management. A medical approach joining high treatment rates with fertility preservation could be a safer and less invasive method of therapy.

We present a case of CSP with a viable embryo that was successfully treated with a novel medical therapy combining multidose methotrexate (MTX) and mifepristone.

No further additional invasive procedure was required since pregnancy products were dissolved and no major complications were experienced.

Multidose MTX and mifepristone can be considered a safe and effective treatment for CSP.

## INTRODUCTION

An ectopic pregnancy is defined as cesarean scar pregnancy (CSP) when the products of conception are implanted within the myometrium at the point of a cesarean section scar. Previously sustained uterine wounds or the presence of scar tissue is one of the major predisposing factors.^1^

The CSP ratio has increased recently to 1 in 1800 to 2500 pregnancies as a result of higher numbers of cesarean deliveries.^2^ Since CSP is a life-threatening condition with serious and emergency medical complications, several different studies have focused on the “gold standard” of therapy, specifying that uterine evacuation should not be considered as the optimal first option of therapy.^3^

To date, there is no clear consensus for CSP management and treatment. Furthermore, in the literature, different methods are presented regarding CSP treatment.^4^ A medical approach joining high treatment rates with fertility preservation could be a safer and less invasive method.

We present a case report of CSP with a viable embryo that was successfully treated with a novel medical therapy combining multidose methotrexate (MTX) and mifepristone (Mifegyne/Exelgyn, France).

## CASE REPORT

A 31-year-old, gravida 1, para 1 Caucasian woman with a positive pregnancy urine test underwent a routine transvaginal ultrasound (TVUS) at 4 weeks plus 5 days of amenorrhea in which a gestational sac was not definitely identified. Her general physical examination and past medical history were unremarkable. The obstetric history revealed that her previous birth was performed via the cesarean route with 2 layers closure 1 year ago with no further complications noticed after the operation. The patient was clinically stable and signs and symptoms positively correlating to an ectopic pregnancy were absent (Table 1). She received oral instructions to have a new ultrasound taken after 7 weeks of amenorrhea. The β-human chorionic gonadotropin (β-HCG) levels were not counted at this initial visit.

**TABLE 1:**
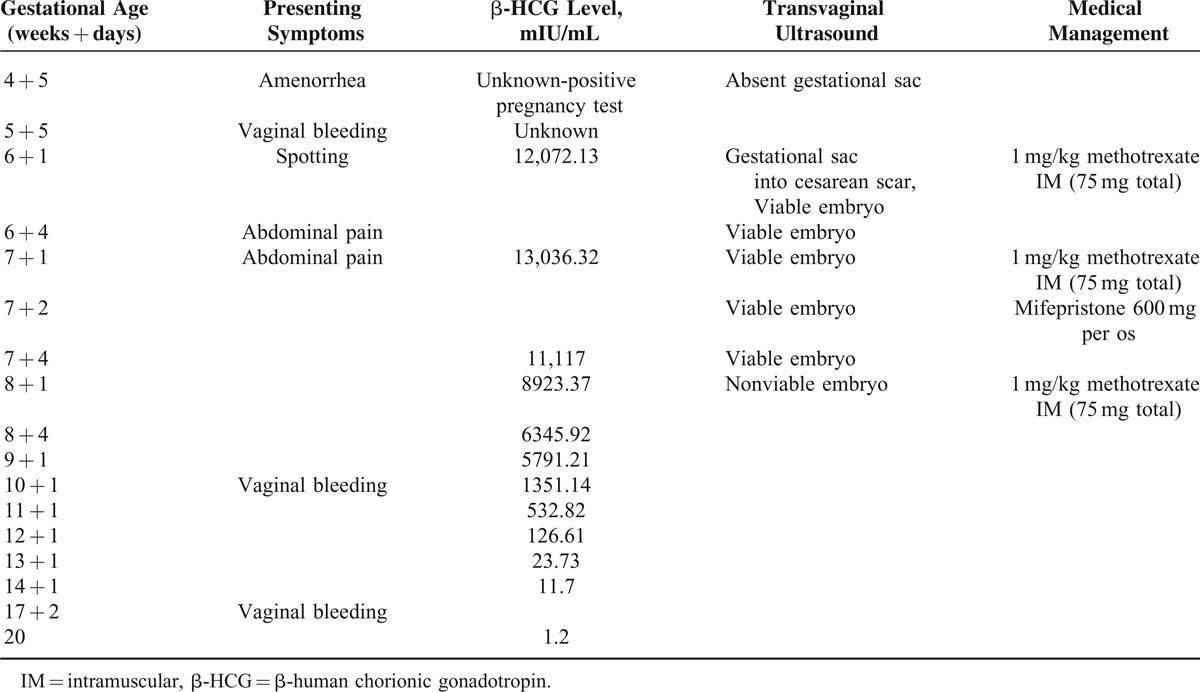
Clinical Data and Chronology of Patient's Evaluation and Treatment

**FIGURE 1:**
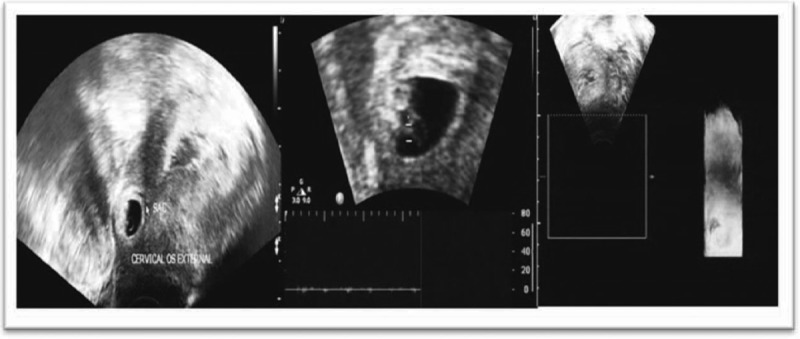
TVUS and 3D TVUS showing a cesarean scar pregnancy gestational sac with a viable fetus located in the anterior part of the isthmic portion of the uterus with diminished myometrial layer between the bladder and the sac. 3D = 3-dimensional; TVUS = transvaginal ultrasound.

At 6 weeks plus 1 days of amenorrhea, she reported vaginal bleeding and underwent a new TVUS and 3-dimensional ultrasound that showed a suspected SCP with a viable fetus (positive fetal heart rate). Ovaries appeared normal and the pouch of Douglas had no fluid (Fig. 1). The CSP diagnosis was then made using previously established diagnostic criteria: previous history of cesarean section, secondary amenorrhea with irregular vaginal bleeding and increased β-HCG levels, empty uterine cavity and regular endometrial lining, empty cervical canal, and diminished myometrial layer between the bladder and gestation sac.^1^

After being informed about the different treatment options as well as the possible benefits and risks of each method, the patient decided to stop the pregnancy via the medical route using the proposed novel combination of multidose MTX and mifepristone. A written informed consent was obtained from the patient. Baseline serum β-HCG was checked at 12,072.13 mUI/mL (Table 1) and tests for liver, renal function, and a complete blood count were performed.

**FIGURE 2:**
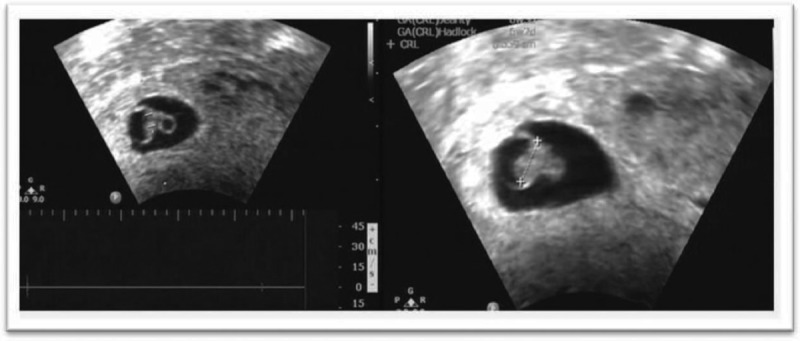
Transvaginal ultrasound (TVUS) demonstrating a nonviable embryo measuring 0.559 cm crown to rump length.

**FIGURE 3:**
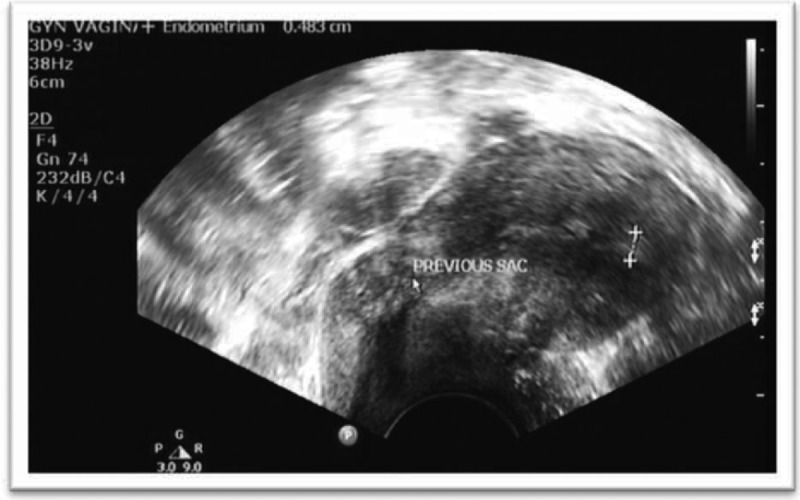
Transvaginal ultrasound (TVUS) demonstrating a normal uterus with no embryonic signs in the place of the previous ectopic sac.

Medical treatment consisted of 1 mg/kg MTX, 75 mg total, intramuscular, on days 0, 7, 14 and mifepristone, *pos*, on day 7. Serum β-HCG was measured every other day, until the titers declined to 50%, and then weekly, until normal levels were reached (Table 1). Also, tests for liver, renal function, and a complete blood count were checked every 3 days. At 8 weeks plus 1 day of gestation, β-HCG decreased to 8923.37 mUI/mL and a TVUS confirmed absence of a fetal heartbeat (Fig. 2). Moreover, resolution of the gestational sac was monitored with a weekly TVUS. At 20 weeks, no visible gestational signs were noticed (Fig. 3).

## DISCUSSION

Systemic multidose MTX therapy, along with mifepristone, seems to be a safe tool for the treatment of CSP, even for those women with a viable embryo. The use of mifepristone appears to be promising in the treatment of CSP for reducing the total doses of MTX and for hastening the time to embryo death.

A PubMed search using the terms “cesarean scar pregnancy,” “methotrexate,” “mifepristone,” “RU486,” and “viable fetus” showed that, to date, this is the first case that has reported the use of mifepristone in combination with systemic multidose MTX therapy for the treatment of a CSP with a viable embryo.

Clinical data and methods of treatment differ among the obstetric-gynecologic community and guidelines regarding the treatment of CSP are missing. MTX systemic administration, ultrasound-guided local administration of embryotoxins, surgical evacuation, laparoscopic removal, and combined medical treatment are different routes of therapy with positive outcomes^5^ that are mentioned in the literature.

Systemic MTX is the first-line therapy for tubal and cervical ectopic pregnancies and can be administered when gestational age is less than 9 weeks, the embryo size is smaller than 10 mm, the fetus is still no viable, and baseline serum β-HCG are less than 10.000 mIU/mL.^1^ In our case, the patient presented with a positive pregnancy urine test at 4 gestational weeks plus 5 days; in the second clinical visit, her β-HCG was at 12,072.13 mUI/mL and further evaluation of the TVUS revealed a gestational sac within the previous cesarean scar with a viable fetus.

Because of the mass experience gained from the use of MTX in ectopic pregnancies, previous reports show the usual dose of MTX systemic administration at 1 mg/kg responds well in the treatment of CSP.^1^ In our case, we used the above-mentioned dosage of MTX, 75 mg total, intramuscularly, on days 0, 7, 14. On day 7, as the embryo continued to be viable, we used 600 mg mifepristone per os, in combination with MTX. To the best of our knowledge, this is the first time the therapy has been attempted in a CSP with a viable embryo, according to the literature.

In a study from Kutuk et al,^6^ the mean total dose cycles of MTX used for the treatment of CSP with cardiac activity was 5.3 and 6 for those without cardiac activity with short intervals (every other day), whereas, in our case, only 3 weekly cycles were required with the use of mifepristone.

In an extensive review from Timor-Tritsch et al,^2,7^ with data from 751 CSP cases, 331 (44.1%) ended up with complications necessitating further surgical, or invasive, assistance. MTX was cited as the leading cause of additional embryonic growth and the higher complication rate (62.1%) among 32 different treatment modalities. In our case, no further additional invasive assistance was required, since no major complications appeared. It is vital to stress that we did not perform dilatation and curettage or any other surgical intervention after the medical treatment.

In conclusion, successful CSP management and treatment is associated with prompt diagnosis and proper treatment to ensure uterine and fertility preservation, which emphasizes the rationale behind the use of medical treatment. Systemic multidose MTX and mifepristone therapy seems to be a safe tool for the treatment of CSP, even for those women with a viable embryo. The use of mifepristone appears to be promising in the treatment of CSP for reducing the total doses of MTX and for hastening the time to embryo death. However, the efficacy and safety of this method requires further prospective trials with representative samples. Thus, for establishing guidelines for the treatment of CSP, clinicians should individually present their cases.
